# FiNCO farms for knowledge exchange: A Colombian seed for a good Anthropocene

**DOI:** 10.1007/s13280-022-01821-0

**Published:** 2023-01-26

**Authors:** Corina Buendía, Erika Garces, Juan C. Aceros

**Affiliations:** 1 Cra. 38 Nr 52-65, Apto 404, Bucaramanga, Colombia; 2 Grupo de investigación en Población, Ambiente y Desarrollo, G-PAD, Universidad Industrial de Santander, Cra. 27 Calle 9, 680002 Bucaramanga, Colombia; 3 Escuela de Trabajo Social, Universidad Industrial de Santander, Cra. 27 Calle 9, 680002 Bucaramanga, Colombia

**Keywords:** Family agriculture, Knowledge co-production, Landscape stewardship, Resilience thinking, Social–ecological resilience, Transdisciplinarity

## Abstract

Returning to Earth’s safe operating space requires driving social–ecological transformations toward a new human–nature configuration, while navigating changes and creatively dealing with crises. Family farms in the Colombian Andes have been navigating changes by using modalities of integrated management that recognize family interdependence with nature, which illustrates social–ecological resilience on a small scale. We potentiated family farming by creating FiNCO (Farms for knowledge exchange) as a strategy that renews the connection between disciplines, academia and farmers, and the rural and urban sectors, in order to promote knowledge co-production and landscape stewardship. In this sense, FiNCO is considered as a seed for a good Anthropocene. This paper is an invitation to plant FiNCO seeds in different social–ecological contexts and to share those experiences as an ideal way to create abilities to transform the upcoming humanity challenges into opportunities for humanity’s needed transformation.

**Supplementary Information:**

The online version contains supplementary material available at 10.1007/s13280-022-01821-0.

## Introduction

Humans are now the dominant force in shaping the Earth. We refer to this time as the Anthropocene (Crutzen 2002; AWG 2019). Such a human-dominated geologic epoch is crossed by turbulence, extreme transitions, and uncertainty at all scales with severe implications for human societies (Folke et al. 2021). Thus, beneficial futures call for the ability to navigate the dynamics of the Anthropocene (Reyers et al. 2018), while people reconfigure social–ecological systems (O’Brien 2012; Pereira et al. 2018a) through renewed human–environmental interactions, active stewardship, and responsible governance schemes (Walker 2003; Folke et al. 2021).

Due to the intensity and complexity of the forces and dynamics at stake, a question arises on how local actors can deliberately contribute to positive pathways of transformation. The innovations required are systemic (Loorbach et al. 2017) and should emerge through multiple dynamics of co-production (Norström et al. 2020; Chambers et al. 2021) among diverse actors and scales (Westley et al. 2013; Reyers et al. 2018). However, a growing literature highlights the importance of micro-level initiatives triggering social learning and innovation (Westley et al. 2011). At such micro-scale, suitable conditions for experimentation and mechanisms connecting innovation with institutional opportunities are required. In this vein, some authors argue for the creation of “seeds of a good Anthropocene” (Bennett et al. 2016), transformative spaces in which people are active creators of change.

The quest for a “good Anthropocene” is built on the present, by using already available elements (Bennett et al. 2016; Pereira et al. 2018c). The “seeds” are part of these elements. They are initiatives in different realms displaying, with different levels of implementation, new cultural repertoires that tackle aspects of global problems (Pereira et al. 2018a). As part of these initiatives, alternative ways of framing situations emerge, suggesting how a system evolves and justifying interventions (Leach et al. 2010). When collectively adopted as stories of promising imagined futures, they act as narratives of hope (Folke et al. 2021) fostering a sense of agency and coordinated actions (Pereira et al. 2018c).

The seeds are local, but they can grow, replicate, spread, and inspire new initiatives (Pereira et al. 2018b). In combination with other seeds, they could promote transformative pathways (Bennett et al. 2016), making macro-level patterns emerge (Reyers et al. 2018). With this in mind, several examples of seeds in diverse areas have been collected by the “Seeds of a Good Anthropocene” project (Bennett et al. 2016; Pereira et al. 2018b). As part of the seeds, Bennett et al. (2016) mention the efforts of the *Satoyama Initiative* in managing socio-ecological production landscapes and seascapes (SEPLS). This initiative is inspired by a Japanese traditional agricultural landscape (*Satoyama*) with positive impacts on biodiversity conservation and development (Duraiappah et al. 2012).

According to the *Satoyama Initiative*, sustainable management of SEPLS requires strong community involvement in understanding and transforming their landscapes (Saito et al. 2020). In aid of people in such an endeavor, the *Satoyama Initiative* developed a set of 20 Indicators of Resilience to be assessed in participatory ways (Bergamini et al. 2013). Such indicators include attributes of SEPLS related to ecosystem protection, biodiversity, knowledge, governance and social equity, and livelihoods and well-being. The indicators have proven to be useful in different contexts (Dublin and Natori 2020: Martínez Pachón et al. 2020).

In the Colombian Andes, we applied the *Satoyama Initiative* indicators during our involvement in the project “Mainstreaming Biodiversity Conservation and Sustainable Management in Priority SEPLS” funded by the Global Environment Facility (also known as the *GEF-Satoyama Project*). The project was implemented from 2016 until 2019 in 10 tropical countries, with the aim of mainstreaming the conservation of SEPLS while improving human well-being (Dublin and Natori 2020). As a result, we have proposed a participatory and transdisciplinary strategy to identify and strengthen resilient family farms serving as models of landscape stewardship in sustainable directions. The name of such a strategy is FiNCO—*Fincas de Intercambio de Conocimiento* (Farms for Knowledge Exchange).

As similar knowledge co-production initiatives, FiNCO interweaves research and practice (Chambers et al. 2021) and embraces the notion that scientists and non-academic actors can collaborate in addressing emerging challenges in the Anthropocene (Norström et al. 2020). FiNCO’s aims are to develop pluralistic coalitions and capacities to (1) share knowledge and context-based experience between family farmers, (2) establish dialogues between academics, students, and local families to co-produce knowledge and solutions to family farming challenges, and (3) promote landscape stewardship using social–ecological resilience as a framework for analysis and action. In subsequent sections, we reflect on how FiNCO surged and present the main lessons derived from the process. For our reflection, we understand FiNCO as a “seed for a good Anthropocene” (Bennett et al. 2016), a promising experience with the potential to co-produce larger-scale transformations.

### Case study

Colombia’s megadiversity of social–ecological systems enhances and challenges rural development and biological conservation. The country is in the equatorial zone where the Andes Mountain range opens into three chains, creating a strong topographic gradient with a high diversity of climatic and soil conditions. Archaeological evidence confirms that cultures have been coevolving with such landscapes for at least 20 000 years (Castaño-Uribe 2019). As part of such a long-term process, agricultural practices are also coevolving with the landscapes (Schill et al. 2019). Continuing with some of the traditions, family farming in Colombia has been an integrated management system that recognizes people’s interdependence with nature and has also been highly relevant to food production.

Family farms are social–ecological systems navigating changes in the Anthropocene. They show the ability to creatively transform themselves with changing conditions, evidencing their resilience. However, family farms also experience growing pressures that threaten their way of life (Hazell et al. 2010). For many years, research, development, and innovation (R&D) efforts have been focused on increasing economic gain for the country by positioning goods that are globally demanded. Since Colombian farmers must fulfill international requirements, agricultural R&D’s aims have been concentrated on increasing yield, homogenizing quality, and treating crop sanitary problems (OECD 2015). Institutions have been focused on strengthening disciplinary approaches to problems, very often bringing proven “solutions” engineered in northern latitudes. Due to the diversity of social–ecological conditions, monoculture expansion to promote competitiveness threatens both ecological and farmers’ resilience (Díaz et al. 2019). Therefore, Colombia needs to define its own pathways for connecting research and education to promote rural sustainable development while recognizing local conditions, potentials, and challenges.

In 2015, while awaiting the peace agreement to be completed, a group of researchers from the Universidad Industrial de Santander (UIS) and the Corporación colombiana de investigación agropecuria—AGROSAVIA applied for the GEF-Satoyama project. Our proposal had two main objectives (1) to identify existing management strategies reconciling biodiversity conservation with agricultural production and (2) to empower local communities to conserve and share their knowledge for meeting future challenges and inspiring the emerging population of post-conflict farmers in Colombia. As a key aspect of the proposal, we postulated a micro-watershed called “Las Cruces,” located in the municipality of San Vicente de Chucurí, as a Satoyama-like landscape.

**Fig. 1:**
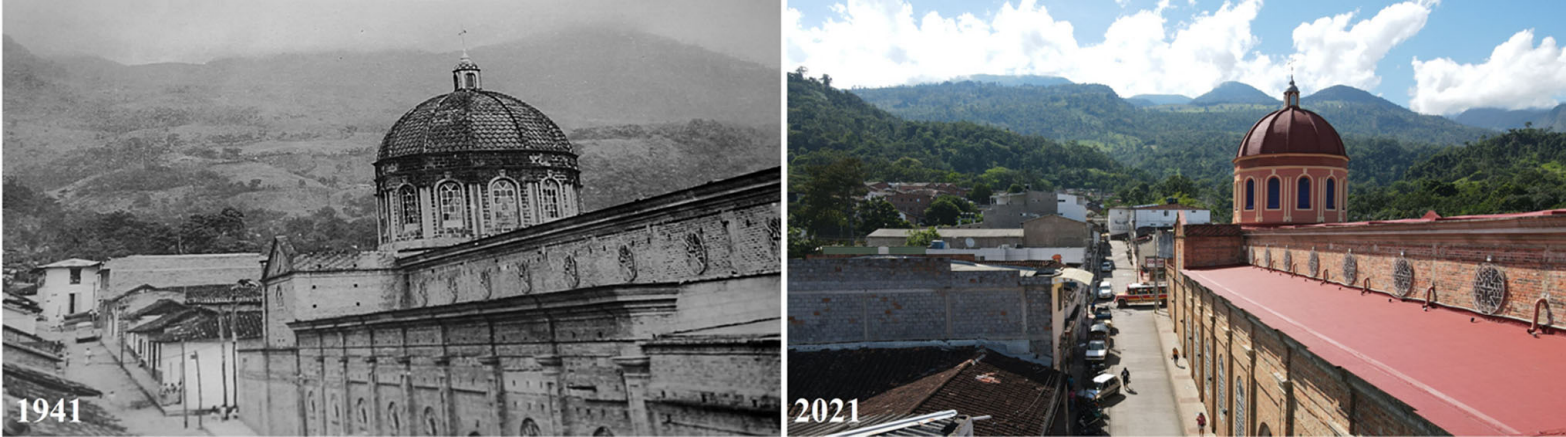
Landscape view over Las Cruces’ micro-watershed, taken from San Vicente de Chucurí Main Square. Left picture in 1941, large-scale cattle production was the dominant land use as few people owned large amounts of land (photo: unknown author). The right picture in 2021 shows the landscapes have more trees and less grass (photo: Noelia Tobalo). Currently, family agriculture is the dominant form of land tenure and agroforestry systems have become dominant

**Fig. 2:**
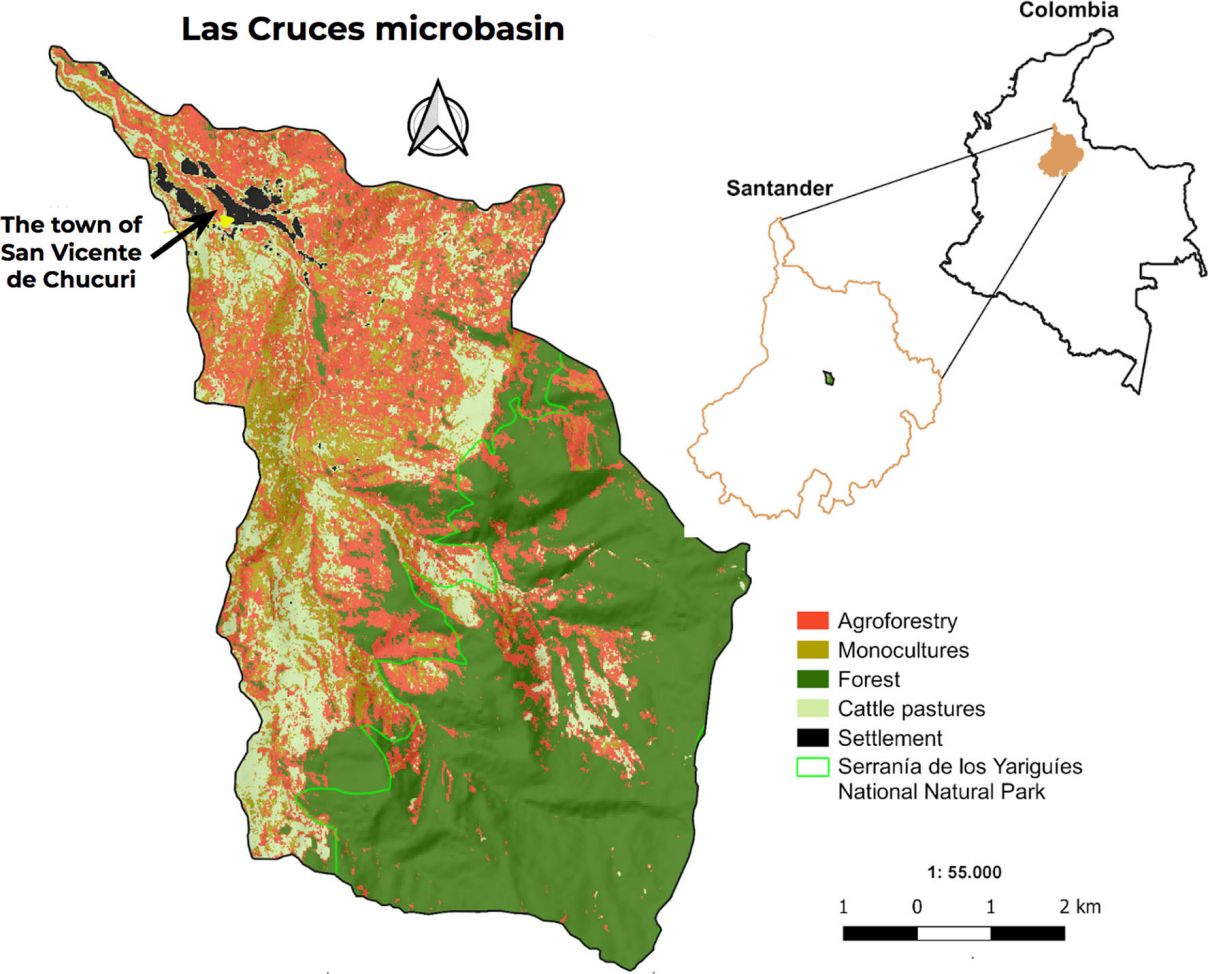
La Cruces micro-watershed land use map estimated in Bolívar-Santamaria and Reu (2021)

San Vicente de Chucurí is a rural municipality in the Department of Santander, located on the western side of the Serranía de Los Yariguíes, a spur of the Eastern Cordillera of the Andes. From far away (Fig. 1, right), this landscape seems like a dense forest, but if one looks closer, one can see a diversified agricultural production (see Appendix S1, Figs. S1 and S2) with high diversity and endemism organized predominantly in agroforestry systems, as shown in the land use map in Fig. 2. The area is influenced by the high humidity regime of the Magdalena River valley and therefore receives significant amounts of precipitation (1600 mm a^−1^) (Pinilla et al. 2018).

The micro-watershed Las Cruces is located in the northeastern part of the municipality. It has an extension of 5779 ha, with a strong topographic and management gradient ranging from forest protected by a National Natural Park (2650 masl) to San Vicente’s town (570 masl). In between, there is a highly productive zone where climate diversity and soil fertility offer diverse possibilities for family farming (see Appendix S1, Fig. S1). Large-scale cattle farming, as well as secondary forest patches, are also present in the area (Bolívar-Santamaría and Reu 2021).

Until the 80s the territory was predominantly dedicated to extensive cattle ranging (Fig. 1, left). The area was immersed in violence and conflict with a strong presence of guerrillas and paramilitary groups from the 1960s until 2005, when the paramilitaries demobilized and the Colombian army took control of the area. Therefore, we considered this territory as a post-conflict model that could inspire transitions toward sustainability in other territories.

## Research strategy

**Fig. 3:**
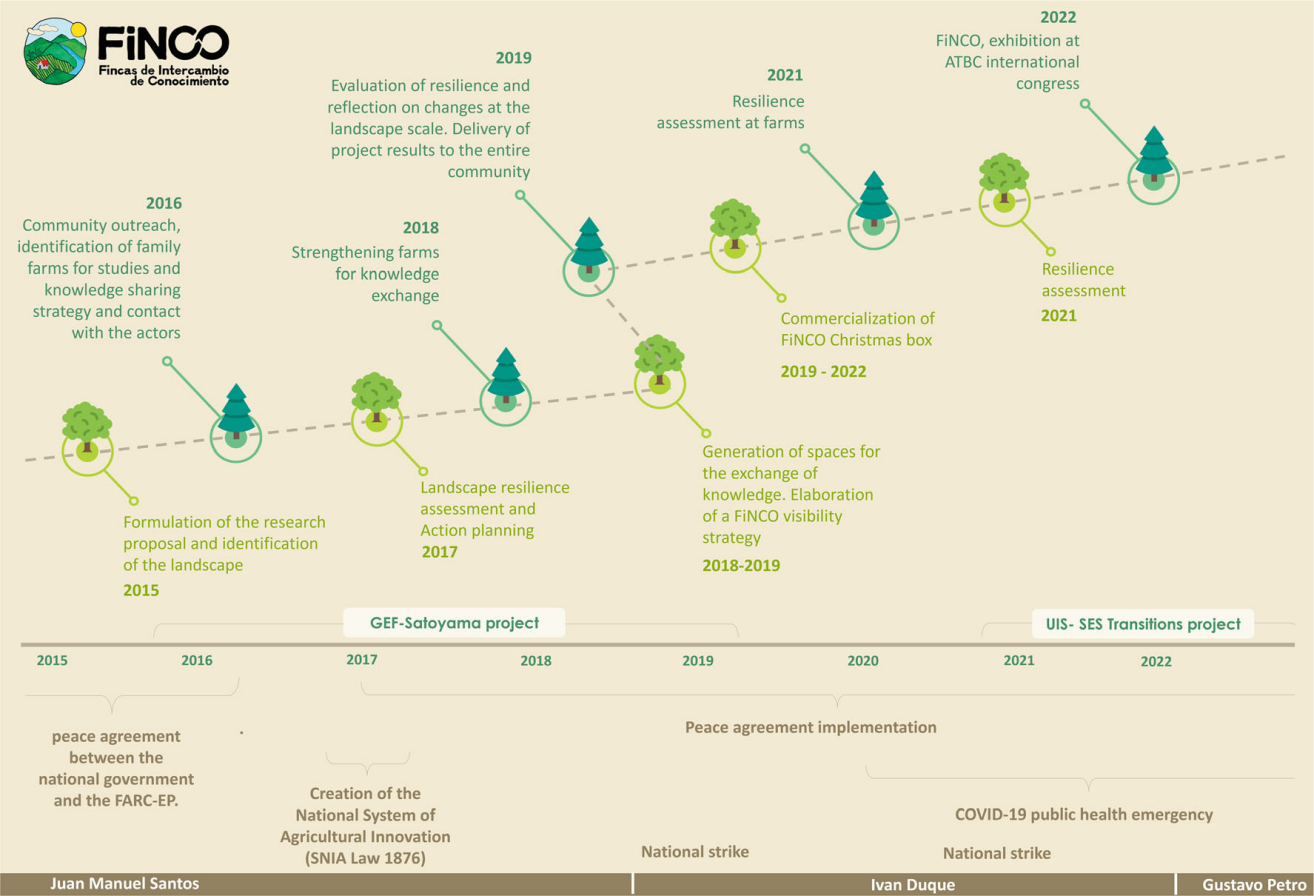
Timeline of FiNCO’s experience. Events and processes from 2015 to present. In green are shown processes that have taken place as we implemented FiNCO. In brown are shown important events and processes that were occurring in Colombia during this time

The core implementation team consolidated key insights gained from the project through personal and collective reflection on designing and implementing FiNCO. The analogy of seeds for a good Anthropocene was used as a framework for the reflection we offer in this paper. We used written project reports, presentations, and interviews to construct the process timeline, support our arguments, and help our memory. We regularly gathered to discuss the origin, implementation, development, and results of FiNCO. The main insights we present in the following sections result from our current consensus but continue to be objects of scrutiny and reflection, as the FiNCO story evolves. Before presenting such ideas, we describe in chronological order the intentions and actions that were performed during the process (see also Fig. 3).

### Implementation of GEF-Satoyama project (2016–2019)

After receiving the GEF-Satoyama grant, we contacted local organizations and, through them, the presidents of the Community Action Boards (JAC, *Juntas de Acción Comunal*) present in Las Cruces. JAC are grassroots organizations involved in decision-making and local management; therefore, we considered their consultation as an essential and respectful step that many NGOs and other institutions omit. JAC’s presidents initially were reluctant to get involved in our project. They told us about previous negative experiences and directly asked us what the community would receive from our project. We sincerely answered that we regarded their landscape as a remarkable example in which agricultural production reconciles with biological conservation and that we wanted to learn how they had made this possible. We put to the disposal of the community our personal and disciplinary knowledge. They liked the idea of academics coming to learn from them. They also mentioned that we could help them to overcome some community challenges, and warmly welcomed us.

With the support of Fundación Natura (a national NGO) and the JAC’s presidents, we visited several farms in the area and selected eight families as allies for knowledge co-production purposes. Additional participants and farms were included to resolve other biological and socioeconomic research questions. To embrace the complexity of the landscape, different disciplines were articulated in the project, including natural and social sciences. During the visits to the territory, it became clear that within the initial research team we had different perceptions about it, and we realized that it was not always possible to reconcile disciplinary research questions with the main goal of the project. It was also evident that students would require additional training aiming to deconstruct preconceptions of farming, local knowledge, and other disciplinary approaches to issues at stake. Therefore, the research seedbed for Social–Ecological Studies was created at UIS as a space for facilitated multidisciplinary dialogue among students and researchers. Approaches from humanities, biology, and social–ecological studies were debated as part of the process.

The *GEF-Satoyama Project* involved the assessment of the Indicators of Resilience in SEPLS (Bergamini et al. 2013) at the beginning and at the end of the intervention (see Appendix S1, Fig. S3). The purpose was to set an initial and final state to document the impact of interventions (see Dublin and Natori 2020). The *GEF-Satoyama project* trained three members of the research team on the application of these indicators and the baseline assessment was realized through two-day participatory workshops. The idea was to facilitate constructive exchanges for the co-production of knowledge about the territory (Drimie et al. 2021) aiming to challenge power dynamics, resource flows, and meaning and values across different contexts and scales (Schäpke et al. 2018; Chambers et al. 2021). Individual questionnaires, social mapping, timelines, interviews, and sociograms provided input for discussion among different family members and local leaders.

The assessment of the indicators led to the identification of SEPLS’ potential and challenges in order to propose actions. The community proposed improving associativity and commercialization chains, increasing knowledge about biodiversity, and establishing community-based touristic offers (Garces et al. 2017). During the following 3 years, severe actions were implemented to support the development of such proposals. For example, members of families were involved in finding the remaining trees of an endemic magnolia species (*Magnolia resupinatifolia*) (see Pimiento-Quiroga et al. 2019). Additional team members documented knowledge about coffee, cacao, and conservation using ethnography, while others documented local biodiversity, and studied the flows of energy and matter within the farms. These activities were possible because making adjustments during the process was allowed by the financing institutions; moreover, the team was able to bring in additional funding.

During the implementation of the project, an increasing need for knowledge-sharing opportunities within and beyond the territory became evident to participants. In 2018, three of the participating families (Fig. 3) expressed deep interest in being part of a pilot on the topic; students and researchers also agreed that those farms were appropriate for that. We decided to name the pilot FiNCO (***Fincas de iNtercambio de COnocimiento***—Farms for knowledge exchange) because it sounds like “Finca” (farm, in Spanish) and since it strengthens the idea that farmers have valuable knowledge to share.

To strengthen the farmers’ capacities to exchange knowledge with others and help them think and plan for the future, an evaluation of the social–ecological resilience was carried out after adapting the *GEF-Satoyama* indicators to the farm scale. The assessment was a space for family intergenerational dialogue where each family created its own plan. With the assumption that valuation increases well-being, we created a marketing strategy for the visibility of those farms, as well as our co-production and landscape management strategy. Design and photography professionals designed a logo and created videos and photographic portraits. Videos presenting the families and their process were created through video-recorded interviews designed by the producers and revisited by each family. Videos and information are available online (Buendía et al. 2019).

In late 2018, we finally formed a transdisciplinary team that worked together to synthesize the project findings, and to position Las Cruces as a SEPL and FiNCO as a strategy for knowledge co-production and rural stewardship. The team included members of the three farms, recently graduated professionals who participated in the *GEF-Satoyama* project, members of the research seedbed, and three of the five researchers who contributed to the initial formulation of the project.

At the beginning of 2019, matching the closing of the *GEF-Satoyama* project, the evaluation of the resilience of the SEPLS was carried out again to reflect on the impacts of the intervention as perceived by the participants (see Appendix S1, Fig. S3). Subsequently, a resilience workshop was held with the participation of institutional actors. In addition, the project’s results, including research outcomes, community action plans, and FiNCO’s strategy, were presented to the community of San Vicente in an interactive format.

### Spreading FiNCO messages (2019–current)

**Fig. 4:**
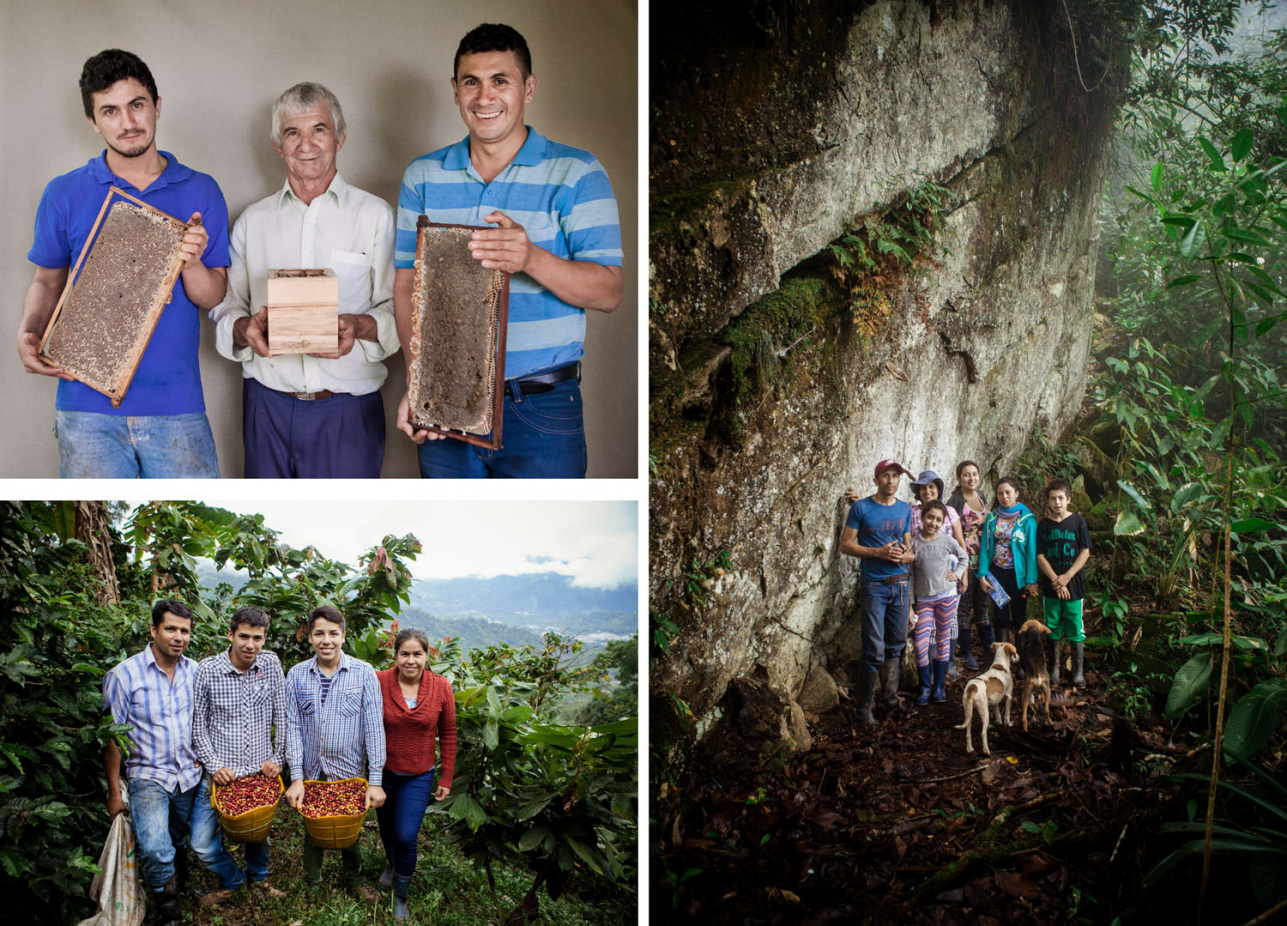
Right picture: the Rodríguez family at *Los Laureles* farm conserved the forest where they have a touristic walking trail. Their farm also offers coffee learning experiences (photo: Noelia Tobalo). Top left portrait: Marcelino Fernández posing with a brood box of native American bees, surrounded by two of his sons, posing with frames of the commercial African honeybee (photo: Noelia Tobalo). Honey represents the main income for their family at *Varsovia*, a 16-ha. diversified farm. Bottom left picture: The Blanco family in their coffee and cacao agroforestry system showing ripe coffee grains from their harvest at their farm *Vista Hermosa* (photo: Noelia Tobalo). Potraits are part of the exhibition Chucureños resilient farmers FiNCO. Stories of the three families are available in Spanish at https://finco.pasoeco.co

**Fig. 5:**
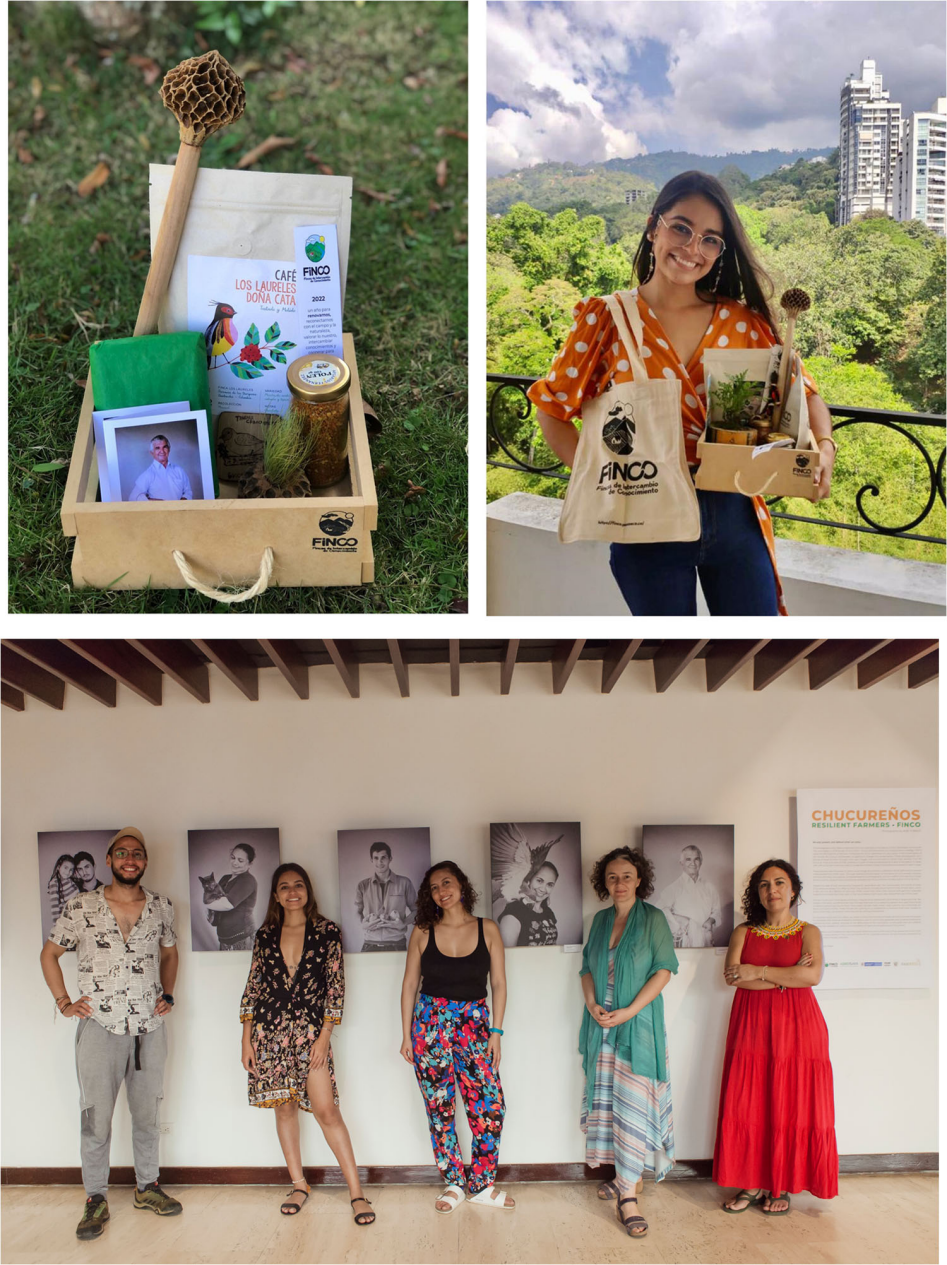
The top pictures show the Christmas boxes containing FiNCO products: coffee, chocolate, honey, pollen, small plants, molinillo (*Magnolia resupinatifolia* wooden fruit), a cotton bag with the FiNCO logo, and a message wishing to increase resilience and rural–urban connections (photos: Corina Buendía). The lower picture shows part of FiNCO’s action-research team posing in front of the exhibition Chucureños Resiliente Farmers FiNCO that was exhibited at the 58th Annual Meeting of the Association for Tropical Biology and Conservation ATBC 2022 that took place in Colombia for the first time (photo: Noelia Tobalo)

In June 2019, we were invited by the photographers’ collective to the event *Encuentro Fotográfico de Santander* to present FiNCO’s portraits in a photographic exhibition and in a forum. The activity sought to highlight the beauty of the countryside and its people (Figs. 4 and 5), showing how intertwined farmers are with their environment. Our intention was to inspire visitors to feel connected with the families of the Colombian countryside. The narratives captured in the videos highlighted how the local way of life has transformed people and their farms, driving this SEPLS toward more sustainable pathways, as can be inferred by the differences between a picture from 1941 and a recently taken one (see Fig. 1). Such a rationale contrasts with many other exhibitions and documentaries in which the victims of Colombia’s internal conflict and its suffering are brought to the forefront.

The refreshing narratives and pictures of FiNCO were also presented in different contexts. In 2022, we brought a renewed version of FiNCO’s exhibition to the 58th Annual Meeting of the Association for Tropical Biology and Conservation, held for the first time in Colombia. The exhibition was there to bring messages of hope to participants: humans can transform our landscapes in a positive way, we can reconcile production with biological conservation, and farmers and academics can co-produce knowledge for sustainability and stewardship (see Fig. 5). We documented congress participants’ impressions about the exhibition by using recorded interviews, some of them available on a YouTube list (Buendía et al. 2022).

As part of the FiNCO initiative, we also started to commercialize Christmas boxes with products and messages about resilience, responsible consumption, and valuation for family farms (Fig. 5). Recently, family farmers decided that they wanted to continue selling the Christmas boxes as this meant both income and recognition for them.

### Implementing UIS social–ecological transitions research project (2021–current)

In May 2021, 2 years after the culmination of the *GEF-Satoyama project*, new research led by UIS started in San Vicente and Zapatoca, a nearby municipality. It is called “Social–ecological transitions in the Northeastern Andes: Connecting knowledge for biodiversity conservation and resilient rural development.” As part of such a project, the resilience assessment of each of the three FiNCO was carried out again. The three families achieved most of their action plans, in some cases with modifications to respond to changing conditions.

The new project includes a systematization of the experience around knowledge co-production that is based on personal and collective reflection after each activity. We have co-created tools for documenting phenology and establishing experimental community settings for the germination and propagation of a species locally known as panelaquemada (*Caryodaphnopsis* sp.). This Lauraceae has fantastic everlasting wood that smells like burned panela when it is cut. This wood is used as constructive material on many farms, but nowadays it is hard to find adult trees. The co-created knowledge and botanical samples around the panelaquemada are now being used for the botanical description of this probably new species for science and for its propagation and conservation.

## Results and discussion

The implementation of the *GEF-Satoyama project* in Colombia allowed the germination of a mixed seed that contained genetic information from Japan and from the researchers who formulated the initial proposal. We planted the seed in Las Cruces and the nascent tree was hybridized with the creativity, visions, and knowledge of farmers, students, and researchers that took part in the process. One of the fruits of this tree is the narrative of resilient family farming that produces seeds of hope. Another fruit is FiNCO as a strategy for knowledge co-production and rural stewardship that inspires positive futures, showing itself as a way forward to strengthen university education and to reconnect the sciences, the rural and urban sectors, and human–nature relationships. In the following sections, we share our reflections on what we considered to be key to the co-production of those fruits. Firstly, we present the role of family farms as seeds for a good Anthropocene. Secondly, the importance of valuing family farming in rural–urban connections. Thirdly, the relevance of moving from a multidisciplinary dialogue toward co-production. Lastly, the contribution of resilience thinking to landscape stewardship.

### Family farms as seeds for a good Anthropocene

Family farming involves the use of the same space for living, producing, and training. Food production for self-consumption or for sale requires integrated management strategies, savings, and investments in productive systems. It also requires recognizing the skills and needs of family members so that each one can contribute to the farm, while the farm contributes to each family member’s life. As a family farmer, the ability to resolve problems with whatever is at hand is essential. The people of Las Cruces are more than 3 h away from the closest hospital and 30 min by car (or about 1.5-h walking time) from the main town. Therefore, they usually have basic plumbing, and animal and human first aid. In order to access essential services, they engage in the construction and maintenance of roads, aqueducts, and schools. All of this requires self-organized community work; therefore, it is usual that their understanding and commitment to the territory goes beyond the limits of their farms and imply the stewardship of the SEPLS. The landscape's historical transformation is evidence of that.

The idea of Las Cruces as a pristine forest being cleared to increase cacao and coffee crops was widespread among environmental authorities and schoolteachers, and perpetuated a misconception that farmers are destroying the environment. However, we soon discovered a different story. From the 1970s to the 1990s, large livestock farms were predominant in the territory (Fig. 1). Since the armed conflict displaced the former landowners, family farming gained space to consolidate. With scarce resources, some inhabitants took advantage of the local conditions and transformed the landscape into a much more diversified SEPLS.

Farmers transformed a savanna into a diversified polyculture system that supports high biodiversity, gaining wisdom within this process. They also evolved different life strategies to more sustainable and resilient ones. The three families involved in FiNCO are our well-documented examples of these positive transformations, but similar stories can be found in Colombia (Rivera et al. 2013; Vallejo-Cabrera et al. 2020; Rodríguez-Suarez et al. 2021). Understanding their stories provides hope for a good Anthropocene. They challenge mainstream narratives about farmers as “victims” (Suarez et al. 2018; Kawarazuka et al. 2022) that deserve our compassion or as “ignorant people” destroying the environment with bad agricultural practices (Robson and Berkes 2011; Sinthumule and Mashau 2020). Therefore, we propose local family farms as a “seed for a good Anthropocene” as they illustrate how such nature–human integrated management systems are able to resist, adapt, and transform in the fate of changes and crises.

### Valuing family farming in rural–urban connections

Becoming a FiNCO has been an inspiring co-production process for the families involved. For example, E. Hernandez (personal communication, August 2, 2021) said: “FiNCO inspired us, for example, to make our own chocolate. Since we made the first one, we never bought chocolate again.” In the same vein, J. Blanco (personal communication, August 2, 2021) expressed: “We want to have a chocolate sales location. I put a lot of emphasis on that (…) because we are FiNCO. Anyone [who knows about the initiative] can say: I want to go there to see what it’s like.”

To support family farmers’ efforts in conserving their SEPLS while finding new livelihoods, stronger connections between rural and urban areas were explored. Selling Christmas boxes (Fig. 4) was a successful strategy in this regard. With their commercialization, we aimed at creating awareness about the production chain and at adding value to the products that support families driving those transformations. As a result, messages of a positive transformation toward sustainability were spread. Additionally, extra income and positive feedback from consumers encouraged the families to go forward and value more what they have and who they are. The following excerpts illustrate this situation:

*I have come to love the trees more with Corina and the team of students she has brought with her. They have taught us a lot of things and this way one appreciates more the things one has on the farm*.

Joaquín Blanco (personal communication, October 14, 2018)

*The students, the teachers made us see the richness we have, they made us love the landscape more. They are giving us wings, and we want to fly with them*.

Esmeralda Rodríguez (personal communication, October 13, 2018)

*Our wings are so well-fitted that we are now flying on our own*.

Esmeralda Rodríguez (personal communication, August 5, 2021)

Along with the commercialization strategy, our own relation with family farms was a crucial ingredient in contributing to alternative connections between rural and urban sectors. FiNCO encouraged the emergence of new narratives in local farmers and, in return, they contributed to our transdisciplinary dialogue. They taught us a pluralistic way to investigate the landscape. FiNCO was also our school and therefore, its team continues to believe and contribute to the landscape and to use this narrative of change for inspiring changes elsewhere. The team works beyond San Vicente and continuously expands, adding new members to reflect on the present, the past, and the future to navigate changes. FiNCO’s videos, portrait exhibitions, and forums contributed to spread family farms as seeds for a good Anthropocene in places far away from San Vicente. Such communicative strategies were very important for creating our own Colombian narratives of positive transformations, essential for a country that is aiming toward sustainability and well-being in the Anthropocene.

### From a multidisciplinary dialogue toward knowledge co-production

FiNCO taught us that investigating and intervening in SEPLS requires an integrative vision, breaking the traditional gaps between natural and social sciences, and between scientific–academic and local knowledge. That is, FiNCO encouraged us to generate action-oriented knowledge that intertwines with multiple forms of knowledge (Pereira et al. 2018c; Drimie et al. 2021). The research seedbed for social–ecological studies (SSE) was essential for making this happen.

A “research seedbed” is a strategy for research learning consisting to groups of students inquiring together about a topic, with the support of professional researchers. In our project, the SSE was a space for participants to discuss challenges, concepts, methods of sustainability science, and ways of intervening in SEPLS. This was not an easy process to carry out; in the beginning, there was a marked division among the participants. Overestimation of their own disciplines as well as ignorance and devaluation of other disciplines and traditional knowledge predominated. The following are some of the ideas that we sought to deconstruct in the seedbed:

1. Farmers are uneducated people who live in poverty and have been abandoned by the state. Our goal is to help them get out of the condition of poverty and fight against injustice and abandonment by the state.
2. The social sciences and humanities do not build scientific knowledge. Only rigorous measurement, with quantitative indicators, offers useful knowledge for biodiversity conservation.
3. The farmers with their bad practices are destroying the environment. We are going to show them how important all living creatures are, and they will feel inclined to stop depredating nature.

Debates were sometimes taken personally, and during this process not all of us were able to be part of the pluralistic approach. Nevertheless, the process within the SSE prompted us to reflect on how we learn and relate to others’ knowledge; it tested our creative, flexible, and resilient capacity, which is a commitment to plural knowledge (Perez et al. 2013). With time, the SSE effectively aided us to identify the above-mentioned ideas as prejudices and to overcome them while enhancing a more integrative approach, which aligned the team to values and beliefs that inspired a renewed form of research and intervention. As an output of our discussions, we outlined a renewed vision as follows:

1. Resilient family farms can be identified in Colombia. We can find families in rural territories that reconcile conservation with agricultural production. Some of them have sorted out all kinds of challenges and have been renewed throughout such a process, evidencing their resilience.
2. Periodic and participatory resilience assessments promote adaptive management. Interactive settings are required for the co-production of knowledge. In this sense, participatory workshops, illuminated by social–ecological resilience, were considered effective spaces for pluralistic dialogue, allowing a shared understanding of the landscape and the implementation of actions toward the increase of resilience.
3. Emerging narratives are promises of a better common future. Successful stories of transformation and family resilience can inspire sustainable transformations. Spreading those narratives opens opportunities for family farmers to be recognized by others as knowledgeable stewards of their landscapes and provides hope for a better collective future.
4. Valuation of family farming, in the form of creative ways of commercializing of local sustainable products, increases people’s well-being. On the one hand, consumers feel they are getting nutritious, loving, and healthy goods that are supporting families capable of reconciling production with biodiversity. On the other hand, families connected through social networks receive positive feedback about their lifestyle, products, and knowledge while receiving a fair price for their products. Fair prices will be achieved if consumers value and know the life stories and production–conservation practices of the families that are behind the production.
5. Farmers can inspire other farmers. Narratives and knowledge exchange of resilient family farms can inspire and guide other families to reconcile production with conservation.

We noticed that most of the students and researchers who worked closely with family farmers and other disciplines were more open, engaged, and more likely to be part of the co-production process. In FiNCO, students, academics and family farmers worked together to co-produce knowledge and landscape stewardship. It was usual for the research team to stay in participants’ households during fieldwork, as well as to be involved in day-to-day activities in the farms, in which informal but informative knowledge exchanges occurred spontaneously. In general, such interactions enhanced appreciation and respect toward the different forms of knowledge, including experience-based ones.

The research seedbed and our ethnographic-like approach were most important for moving from transdisciplinarity toward co-production of knowledge. Norström et al. (2020) proposed four general principles underlying high-quality knowledge co-production for sustainability research: *context-based, pluralistic, goal-oriented, and interactive*. We understand FiNCO as an initiative fulfilling each of these principles. As we have just mentioned, understanding and promoting social–ecological resilience at Las Cruces provided the context and the key place to come to a pluralistic process that was supported by farmers, researchers, and students. Positioning products, services, and narratives required articulating ideas, testing, hearing clients, making multiple adjustments, bringing multiple abilities together for production, persuading, and selling. The project had a general initial goal, but it was only after the participative evaluation of resilience that more specific goals were defined together with the community. The flexibility of the grant and the willingness to support community action plans encouraged some of the researchers and farmers to leave their comfort zone, to learn new concepts, and to work together toward specific aims. The process was goal-oriented, and we were able to assemble a more pluralistic team that followed the main objective. The first phase of the process was very interactive and involved students and researchers visiting the farms for workshops, ethnographic tasks, surveys, and biological data collection. During the time without funding, the goal of making the Christmas boxes and spreading FiNCO’s positive messages promoted constant communication between FiNCO members, even during the course of the pandemic. FiNCO can be strongly identified with three (out of the eight) approaches for co-production defined in Chambers et al. (2019): relating together, empowering marginalized actors, and shaping direct agency.

### Resilience thinking to promote landscape stewardship

Resilience thinking includes understanding the social–ecological context from diverse perspectives and proposing strategies for adaptation and transformation under changing conditions. The resilience workshops we employed as part of the FiNCO initiative provided a platform for farmers’ mutual recognition, learning, and sharing of knowledge about their landscape, its transformation, and future actions to improve different aspects of it. Facilitators played a key role in maintaining a good and productive flow during meetings. During the workshops, local communities were able to enrich the implicit narrative about their landscape and agency, and researchers and students received a valuable tour of the landscape through the community’s stories, opinions, and explanations. Therefore, the created dialogue turned into some of the students’ projects being directed toward resolving specific needs, including documentation of knowledge, improving economic return, creating an agroecological touristic offer, and documenting local biodiversity. Most of the plans were implemented. Farmers were empowered by participating in these spaces, learned the methodology and the main concepts, and gained a new perspective on the history of their territory and its present configuration; based on that, they proposed plans to move forward.

The resilience workshop (2019)—to which relevant stakeholders were invited—resulted in farmers sharing narratives of their SEPLS’ transformation. Their participation in different resilience planning workshops and our action-research projects gave them confidence and academic material to present the transformation they carried out. Farmers, as they better understand their agency in the SEPLS, started to question the role of the regional environmental authority. Therefore, the workshop was an effective dialogue space that promoted common understanding and negotiation between farmers and relevant stakeholders. Also, participants gained a broader perspective of the territory and made personal contacts with farmers and other institutions. Hence, environmental authorities and NGOs can now plan interventions taking into consideration the new comprehension of the territory while involving community members in their formulations. Considering those dynamics we think that resilience thinking was able to promote landscape stewardship at Las Cruces.

## Concluding remarks: FiNCO as a seed for a good Anthropocene

The Anthropocene is a geologic epoch characterized by expected and unexpected changes, some of which might cause the destruction of different social–ecological systems, breaking institutions and markets, and finally releasing space and resources (Pereira et al. 2018c; Folke et al. 2021). The emerging new configuration of the system will be reorganized depending on the seeds that have been previously planted there. New emerging conditions, abilities, and knowledge might enable seeds for a good Anthropocene to sprout to renew values, institutions, and markets, driving a reorganization of the system that promotes planetary stewardship (Bennett et al. 2021)

FiNCO is one of the ripe fruits of the GEF-Satoyama-Colombian tree. With this paper, we illustrate the seeds of this valuable fruit by sharing our reflections and learnings on how we germinated and took care of the tree. FiNCO evidences a way to connect research, education, and rural development. It offers a path to consolidate products and services that value and promote local, cultural, and biological diversity while increasing local capacities and connections to larger scales. The initiative facilitates a transdisciplinary dialogue for territorial innovation that considers current well-being, bio and agrobiodiversity, resources management (natural and economic), innovation and knowledge, and governance and equity. All these elements enhance the abilities of farmers, but also those of researchers, and encourage students to participate in knowledge co-production, making it possible to consolidate a coalition at the landscape level with the potential to improve already existing capacities, in order to navigate local and global changes. In this sense, FiNCO is a strategy for knowledge co-production that promotes landscape stewardship.

FiNCO’s seeds are now ready to be planted in other social–ecological contexts as a way to increase our capacities to deal with changing conditions and drive transformations at landscape levels. Taking care of each seedling will have its own context-specific, complex, and diverse challenges. Counting on the diversity of knowledge and abilities will be of great help if team members can appreciate other opinions and views, believe in the importance and values of FiNCO, and collaborate toward more sustainable futures. The social–ecological context and the appropriate care might allow the seed to become a tree of knowledge co-production that promotes stewardship at the local scale. Capturing the learnings and insights gained from each attempt to plant a FiNCO tree will help us acquire understanding and create the capacities necessary to upscale FiNCO for driving changes at larger scales. Therefore, we invite you to plant a FiNCO seed and share your learnings to spread hope. Investing now at local scales in these novel forms of valuing each other, connecting with the environment, learning to collaborate, creating fair value changes, co-producing knowledge, and promoting local stewardship is the best way to prepare for climate change and other global changes.

Let us start planning now so that we can harvest in our better common future in the Anthropocene!

## Supplementary Information

Below is the link to the electronic supplementary material.

Supplementary file1 (PDF 423 kb)
