# Supplementary material for: FiNCO farms for knowledge exchange: A Colombian seed for a good Anthropocene
Source: Ambio. 2023 Jan 26;52(5):963–75. doi: 10.1007/s13280-022-01821-0 (PMC10073398; doi:10.1007/s13280-022-01821-0)
Supplement: Supplementary file 1 — Supplementary file1 (PDF 423 kb) [file 13280_2022_1821_MOESM1_ESM.pdf]

Ambio

Supplementary Information

This supplementary information has not been peer reviewed.

Title: **FiNCO Farms for knowledge exchange: A Colombian seed for a good Anthropocene**

S1

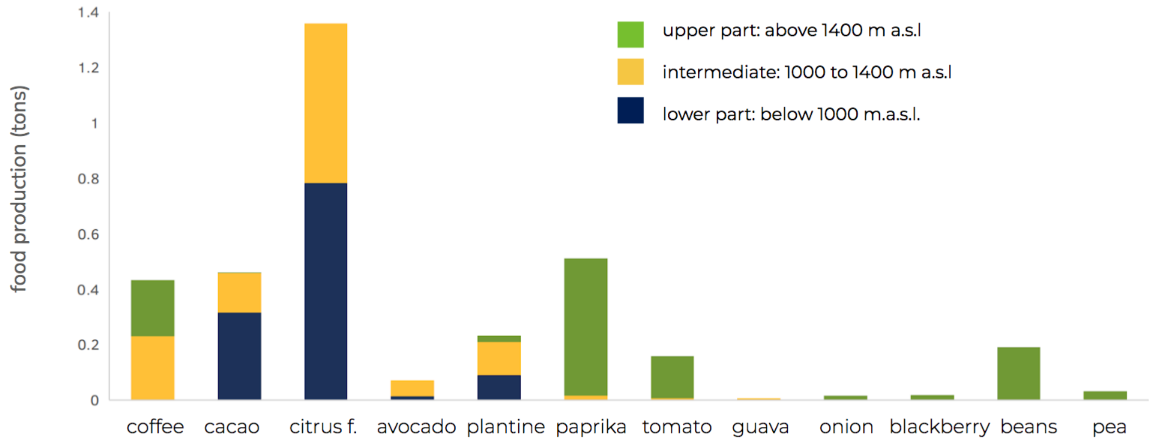

Figure S1. The bar chart shows the total food production for Las Cruces micro-watershed estimated from a socioeconomic survey applied in 2018 using estimates for 2017. Results were divided by altitudinal range in order to see production differences at the altitudinal gradient represented in this mountain social-ecological system. The survey was part of a master's thesis that was embedded in GEF-Satoyama project (Rodriguez, 2018).

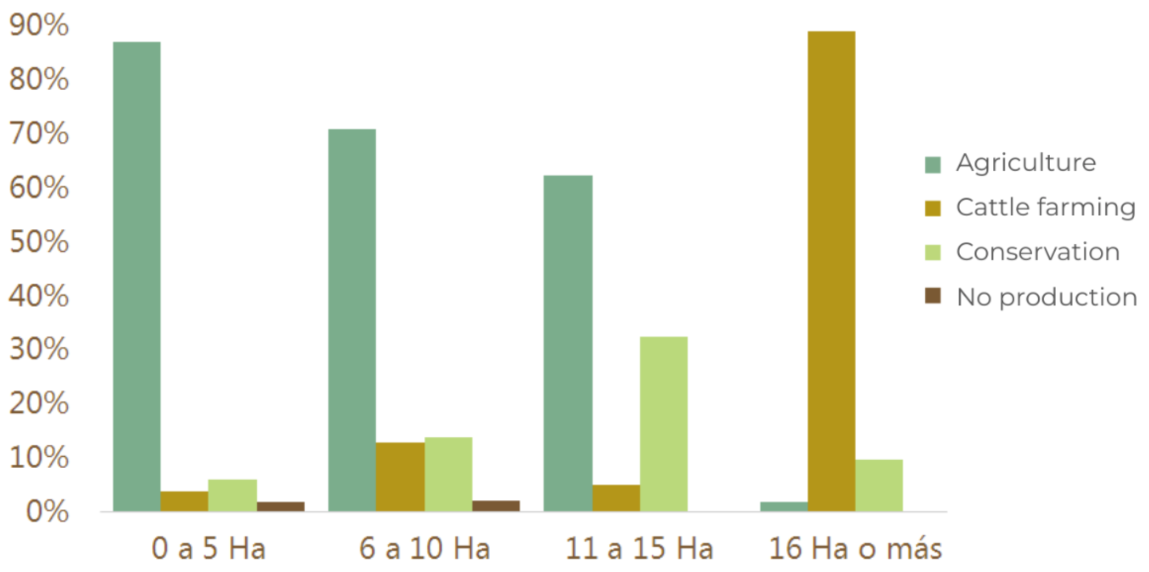

Figure S2. This bar chart shows the average farmland use distribution for farms of different sizes. The survey was part of a master's thesis that was embedded in GEF-Satoyama project (Rodriguez, 2018).

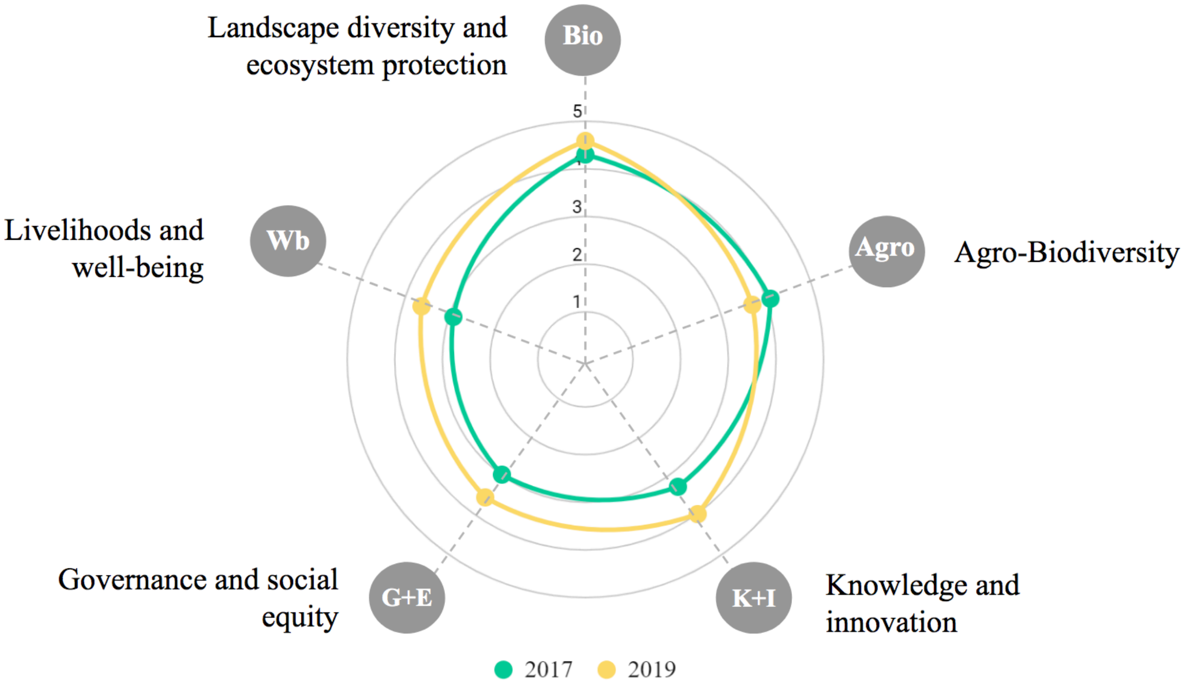

Figure S3. Results from the resilience evaluation of Las Cruces SEPL at the beginning 2017 and at the end 2019 of the GEF-Satoyama project implementation. The evaluation were done using and adaptation from Bergamini et al., 2013 and the idea presented in Natori and Dublin 2019 to evaluate the impact of the intervention.

References

Bergamini, N., R. Blasiak, P. Eyzaguirre, K. Ichikawa, D. Mijatovic, F. Nakao, and S. Subramanian. 2013. *Toolkit for the Indicators of Resilience in Socio-Ecological Production Landscapes (SEPLS)*. United Nations University Institute for the Advanced Study of Sustainability.

Dublin, D and Y. Natori. 2020. Community-based project assessment using the indicators of resilience in SEPLS: Lessons from the GEF-Satoyama Project. *Current Research in Environmental Sustainability* 2: 100016. https://doi.org/10.1016/j.crsust.2020.100016.

Rodriguez, T. 2018. Evaluación de impacto ex-ante de la transformación productiva en la microcuenca Las Cruces del municipio de San Vicente de Chucuri: una mirada desde los servicios ecosistémicos. Master Thesis in Economics and Development. Universidad Industrial de Santander, Bucaramanga, Colombia.
